# Dr. Churchill Dead

**Published:** 1878-06-20

**Authors:** 


### DR. CHURCHILL DEAD.

The eminent Obstetrican of Dublin, Ireland, Dr. Churchill, is no more. He died on the 31st of March at Ardtrea, in the county of Tyrone, at the residence of his son-in-law, Rev. Dr. Meade, where he had lived since his retirement from practice in 1875.

The number of obstetrical cases attended by Dr. Churchill, exclusive of abortions, amounted to 2,-547.

AMERICAN MEDICAL ASSOCIATION.—The merican Medical Association is appointed to meet in Atlanta, Ga., first Monday in May, 1879.

